# High levels of tissue inhibitor of metalloproteinase-1 (TIMP-1) in the serum are associated with poor prognosis in HPV-negative squamous cell oropharyngeal cancer

**DOI:** 10.1007/s00262-019-02362-4

**Published:** 2019-06-25

**Authors:** Timo Carpén, Timo Sorsa, Lauri Jouhi, Taina Tervahartiala, Caj Haglund, Stina Syrjänen, Jussi Tarkkanen, Hesham Mohamed, Antti Mäkitie, Jaana Hagström, Petri S. Mattila

**Affiliations:** 1 Department of Otorhinolaryngology-Head and Neck Surgery, University of Helsinki and HUS Helsinki University Hospital, P.O.Box 263, 00029 HUS Helsinki, Finland; 2 Department of Pathology, University of Helsinki and HUS Helsinki University Hospital, P.O.Box 21, 00014 HUS Helsinki, Finland; 3 Department of Oral and Maxillofacial Diseases, University of Helsinki and HUS Helsinki University Hospital, P.O.Box 41, 00014 HUS Helsinki, Finland; 4 Department of Oral Diseases, Karolinska Institutet, Huddinge, Sweden; 5 Department of Surgery, University of Helsinki and HUS Helsinki University Hospital, P.O.Box 440, 00029 HUS Helsinki, Finland; 6 Research Programs Unit, Translational Cancer Biology, University of Helsinki, P.O.Box 63, 00014 Helsinki, Finland; 7 Department of Oral Pathology and Oral Radiology, University of Turku, Lemminkäisenkatu 2, 20520 Turku, Finland; 8 Department of Pathology, Turku University Hospital, Kiinamyllynkatu 10, 20520 Turku, Finland; 9 Division of Ear, Nose and Throat Diseases, Department of Clinical Sciences, Intervention and Technology, Karolinska Institutet and Karolinska Hospital, 171 76 Stockholm, Sweden; 10 Research Program in Systems Oncology, Faculty of Medicine, University of Helsinki, Helsinki, Finland

**Keywords:** HPV, Oropharyngeal cancer, MMP-8, TIMP-1, Survival, Prognosis

## Abstract

**Background:**

An emerging subset of oropharyngeal squamous cell carcinomas (OPSCC) is caused by HPV. HPV-positive OPSCC has a better prognosis than HPV-negative OPSCC, but other prognostic markers for these two different diseases are scarce. Our aim was to evaluate serum levels and tumor expression of matrix metalloproteinase-8 (MMP-8) and tissue inhibitor of metalloproteinase-1 (TIMP-1) and to assess their prognostic role in HPV-positive and HPV-negative OPSCC.

**Materials and methods:**

A total of 90 consecutive OPSCC patients diagnosed and treated with curative intent at the Helsinki University Hospital between 2012 and 2016 were included. Serum samples were prospectively collected. An immunofluorometric assay and an enzyme-linked immunosorbent assay were used to determine MMP-8 and TIMP-1 serum concentrations, respectively. HPV status of the tumors was determined using a combination of HPV-DNA genotyping and p16-INK4a immunohistochemistry. The endpoints were overall survival (OS) and disease-free survival (DFS).

**Results:**

High TIMP-1 serum levels were strongly and independently associated with poorer OS (adjusted HR 14.7, 95% CI 1.8–117.4, *p* = 0.011) and DFS (adjusted HR 8.7, 95% CI 1.3–57.1, *p* = 0.024) among HPV-negative patients; this association was not observed in HPV-positive OPSCC. Although TIMP-1 was immunoexpressed in the majority of the tumor tissue samples, the level of immunoexpression was not associated with prognosis, nor did MMP-8 serum levels.

**Conclusion:**

Our results indicate that serum TIMP-1 levels may serve as an independent prognostic marker for HPV-negative OPSCC patients.

## Introduction

The incidence of oropharyngeal squamous cell carcinoma (OPSCC) is increasing in many countries due to infection with oncogenic HPV strains. Currently, more than a half of all newly diagnosed OPSCCs in Northern Europe and in North America are HPV related [1–4]. However, tobacco smoking and heavy alcohol use are still major risk factors for the development of OPSCC, especially HPV-negative OPSCC [5, 6]. Survival and recurrence-free rates of HPV-positive OPSCC are significantly better than its HPV-negative counterparts [6–8]. Therefore, developing management and post-treatment surveillance for these patients (particularly for the HPV-negative subgroup) warrants the search for new prognostic markers.

Previous studies have indicated that matrix metalloproteinases (MMPs) and tissue inhibitors of metalloproteinases (TIMPs) exert an important role in tumor pathogenesis and patient survival in various cancers, including head and neck cancers [9–11]. MMPs are a group of zinc-containing genetically distinct but structurally related proteolytic enzymes that degrade almost all extracellular matrix proteins [12]. These proteins may also have a direct cell-signalling effect on various cell-surface proteins, such as cluster of differentiation (CD)44 and integrins [13, 14]. Additionally, MMPs and TIMPs affect apoptosis, cancer cell growth, and immune surveillance, which in turn can promote invasion and metastasis [9, 15, 16]. Increased expression of certain MMPs can be detected in most human cancers, and their overexpression is associated with poor prognosis [9, 17, 18]. On the other hand, regardless of the active role of various MMPs in tumor progression, matrix metalloproteinase-8 (MMP-8) may have antitumor activity [19–21]. MMP-8 may modulate tumor cell adhesion and invasion by processing non-matrix bioactive inflammatory mediators [21–23]. Thus, the role of MMPs in cancer is very complex. This is also evident based on the finding that high tumoral immunoexpression of tissue inhibitor of metalloproteinase-1 (TIMP-1), which is an inhibitor of various MMPs [24, 25], is associated with poor prognosis in various cancers [26, 27]. These findings are consistent with observations indicating that cancer patients with high TIMP-1 serum levels [11, 27–30] are associated with poor prognosis. This may be explained by the additional ability of TIMP-1, which is an inhibitor of various MMPs, to function as a growth factor by binding to the cell surface ligand CD63 [31, 32]. This binding results in the activation of intracellular focal adhesion kinase (FAK) that can promote cancer progression [33, 34]. Overall, different serum levels of MMPs and TIMP-1 may serve as potential prognostic markers in different cancers.

To the best of our knowledge, the role of MMP-8 and TIMP-1 in OPSCC is unknown. To evaluate their role as prognostic factors, we studied serum levels of MMP-8 and TIMP-1 and their expression in OPSCC tumor tissue. Our specific aim was to study the association of MMP-8 and TIMP-1 with prognosis in HPV-positive and HPV-negative OPSCC.

## Materials and methods

### Patients

Patients with consecutive biopsy-proven OPSCC diagnosed and treated with curative intent during a 4-year time period between March 2012 and May 2016 at the Departments of Oncology and Otorhinolaryngology-Head and Neck Surgery at the Helsinki University Hospital, Helsinki, Finland were included. The inclusion criteria were tumor tissue availability for p16 and HPV DNA PCR status determination from each tumor and collected serum samples at the time of diagnosis from each patient. A total of 90 OPSCC patients met the inclusion criteria.

Clinical data included age on date of OPSCC diagnosis, sex, history of tobacco smoking and heavy use of alcohol, tumor-related factors, the date of treatment completion, and details on follow-up. All the data were collected from medical records and it is partly the same as in our previous reports [35, 36]. Tumor stage was determined according to the 8th edition of the American Joint Committee on Cancer staging [37]. Treatment modalities were dichotomized to radiotherapy with or without cisplatin-based chemotherapy and surgery with or without postoperative (chemo)radiotherapy.

Follow-up time was determined from the date of treatment completion to the date of last follow-up or death. Both follow-up time and follow-up protocol were adopted from our previous report [35].

### MMP-8 and TIMP-1 serum concentrations

Sera were extracted from peripheral blood samples by centrifugation at 1600 g for 10 min and stored at − 70 °C. An immunofluorometric assay [38, 39] and an enzyme-linked immunosorbent assay kit (GE Healthcare UK Limited, Buckinghamshire, UK) were used to determine MMP-8 and TIMP-1 serum concentrations, respectively. All analyses were performed in duplicate. Serum concentrations are shown as pmol/l (pM).

### HPV DNA genotyping

HPV DNA was detected by PCR from tumor tissue samples. Multiplex HPV Genotyping Kit^®^ (DiaMex GmbH, Germany) was used to detect 24 different HPV genotypes. The method detects following low-risk HPV genotypes: 6, 11, 42, 43, 44, and 70, and following high-risk HPV genotypes: 16, 18, 26, 31, 33, 35, 39, 45, 51, 52, 53, 56, 58, 59, 66, 68, 73 and 82. The method is described previously in more detail [36]. For the present study, HPV DNA positivity was summarized as HPV positivity for any high-risk type.

### Immunohistochemistry of p16, MMP-8, and TIMP-1

p16-INK4a status was determined by immunohistochemistry (IHC) on paraffin-embedded formalin-fixed tissue samples. Tissue slides were cut, deparaffinized, and rehydrated according to routine protocol [40]. The treated slides were heated in Tris-HCl buffer (pH 8.5) by PreTreatment module (Lab Vision Corp., UK Ltd, UK). Monoclonal mouse anti-human p16INK4a (9517 CINtec Histology Kit, MTM laboratories, Germany) was used as a primary antibody and p16 expression was considered positive if > 70% of tumor cells were strongly positive, as also described previously [36, 40]. Immunohistochemical staining and Western immunoblot of TIMP-1 were analyzed from tissue microarray (TMA) blocks and Monoclonal Mouse IgG_2B_ (R&D Systems, MAB970, Minneapolis, USA) was used as a primary antibody [41]. Specific polyclonal rabbit anti-human MMP-8 [38, 42] was used as the primary antibody for MMP-8 immunohistochemical staining of the TMA blocks and to analyze the molecular forms of MMP-8 by Western immunoblot. Western blotting for both TIMP-1 and MMP-8 were performed by the ECL-Western blotting analysis system as described earlier [42]. The proteins in the SDS-PAGE-gels were treated by electrophoresis, followed by membrane reaction with the primary antibody (1:500) after the proteins were first electrotransferred onto a nitrocellulose mebrane (Bio-Rad Laboratories, Richmond, California) and blocked with 3% gelatin as previously described [42]. Alkaline phosphatase conjugated antibody was used for secondary immunoreaction with the proteins and the final quantitation was performed by a densitometry (Bio-Rad Model GS-700 Imaging Densitometer, Hercules, CA) after the proteins were first visualized using 5-bromo-4-chloro-3-indonyl-phosphate disodium salt (Sigma) and nitro blue tetrazolium (Sigma, St. Louis, Missouri) [42, 43]. Gingival tissue was used as a positive control for both MMP-8 and TIMP-1. A slide in diluent without primary antibody in each immunostaining served as a negative control.

### Immunoscoring

TMA slides were independently evaluated and scored by two researchers (Timo Carpén and Jaana Hagström). Each tumor had six 1-mm punches scored and the slides were re-scored in case of discrepancy. Tumor and stromal cells were scored separately. The scoring of TIMP-1 and MMP-8 in tumor tissue was assessed as follows: negative (0), mild positivity (1), moderate positivity (2) and strong positivity (3). MMP-8 and TIMP-1 scoring in the inflammatory cells were assessed regarding to the number of positive cells as follows: negative (0), 1–20 positive cells (1), 20–100 positive cells (2) and > 100 positive cells (3).

### Statistical analysis

All statistical analyses were performed with IBM SPSS Statistics 25 (IBM, Somers, IL, USA). The endpoints were overall survival (OS) and disease-free survival (DFS). OS was defined as the time from the completion of treatment to death from any cause. DFS was defined as the time from the completion of treatment to the first recurrence or death from any cause. A Cox proportional hazard model was used to evaluate hazard ratios (HRs) in the univariate and multivariate setting. To improve HPV specificity without affecting the sensitivity the combined HPV status of p16 and HPV DNA PCR was used as previously highly recommended [44, 45]. Tumors that are both HPV DNA positive and p16 positive were classified as HPV-positive (HPV +); and the other combinations were classified as HPV-negative (HPV −) as defined in previous reports [44, 46]. Logarithmic transformations were applied for MMP-8 and TIMP-1 serum concentrations to eliminate positive skewness. Survival curves were drawn using the Kaplan–Meier estimate and the statistical significance was calculated with the log-rank test. Receiver operating characteristic curves were used to assess the optimal cut-off serum concentration to discriminate patients with favorable and unfavorable survival. The value that maximizes Youden index (sensitivity + specificity-1) was chosen as an optimal cut-off. A comparison of medians of continuous variables with categorical variables was performed using Mann–Whitney *U* test and Kruskal–Wallis test when suitable. The two-sample *t* test was used to compare means of normally distributed continuous variables between two independent groups. Chi-squared and Fisher´s exact tests were used for categorical data cross tabulation. A two-sided *p*-value < 0.05 was considered as the level of significance.

## Results

### Patient characteristics

Of the total 90 patients, the majority (*n* = 66, 73.3%) were male. Mean age was 61.8 years (range, 36.6–84.7 years). Sixty-six (73.3%) of the tumors were p16 positive and 24 (26.7%) were p16 negative. Fifty-five (61.1%) tumors were HPV DNA positive. The detected high-risk HPV genotypes were as follows: HPV16 (*n* = 51, 92.7%), HPV18 (*n* = 1, 1.8%), and HPV33 (*n* = 3, 5.5%). Fifty-three (58.9%) tumors were both p16 positive and HPV DNA positive and were considered as HPV positive. The remaining 37 (41.1%) tumors were classified as HPV negative and they included the following combinations of HPV and p16 status: p16-/HPV DNA- (*n* = 22, 24.4%), p16 +/HPV DNA- (*n* = 13, 14.4%), and p16-/HPV DNA + (*n* = 2, 2.2%). Smoking, heavy alcohol consumption, and advanced stage disease (III–IV) were significantly more common among patients with HPV-negative OPSCC than among patients with HPV-positive OPSCC. Patient characteristics and tumor-related factors are presented in Table 1.

**Table 1:** Clinicopathological data and TIMP-1 and MMP-8 serum concentrations and immunoexpressions according to HPV status

Variable	HPV +	%	HPV −	%	*p*-value
Number of patients	53	58.1	37	41.9	
Mean age at diagnosis	61.6		62.2		0.755
*Sex*					
Male	43	81.1	23	62.2	
Female	10	11.1	14	37.8	0.045*
*Smoking*					
Non-smoker	20	37.7	8	21.6	
Ex-smoker	24	45.3	6	16.2	
Current smoker	9	17.0	23	62.2	< 0.001**
*Heavy alcohol use*					
Never	28	70.0	15	44.1	
Former	2	5.0	8	23.5	
Current	10	25.0	11	32.4	0.028*
*T class*					
T1–T2	35	66.0	23	62.2	
T3–T4	18				
T2	34.0	14	37.8	0.705	
*N class*					
N0–N1	50	94.3	24	64.9	
N2–N3	3	5.7	13	35.1	< 0.001**
*Stage*					
I-II	44	83	19	51.4	
III-IV	9	17.0	18	48.6	0.001**
*Grade*					
I	1	1.9	2	5.4	
II	3	5.7	12	32.4	
III	49	92.5	23	62.2	< 0.001**
*Treatment*					
(C)RT	39	73.6	23	62.2	
Sx +− (C)RT	14	26.4	14	37.8	0.249
TIMP-1 serum level (pM)	8206		7869		0.879
MMP-8 serum level (pM)	762		844		0.253
*TIMP*-*1 immunoexpression*					
0	7	13.5	6	18.8	
1	30	57.7	20	62.4	
2	15	28.8	6	18.8	
3	0	0	0	0	0.541
*MMP*-*8 immunoexpression*					
0	3	5.9	4	12.5	
1	34	66.7	13	40.6	
2	13	25.5	11	34.4	
3	1	2.0	4	4.8	0.052

(*C*) chemo, *MMP-8* matrix metalloproteinase-8, *RT* radiotherapy, *Sx* surgery, *TIMP-1* tissue inhibitor of metalloproteinase-1

Serum levels of TIMP-1 and MMP-8 are as presented as mean concentrations. TIMP-1 immunoexpression was scored from the tumor tissue. MMP-8 immunoexpression was scored from the inflammatory cells adjacent to the tumor tissue. *p* < 0.05*, *p* < 0.01**

### TIMP-1 is immunoexpressed in the majority of the tumors and MMP-8 in the surrounding cells

**Fig. 1:**
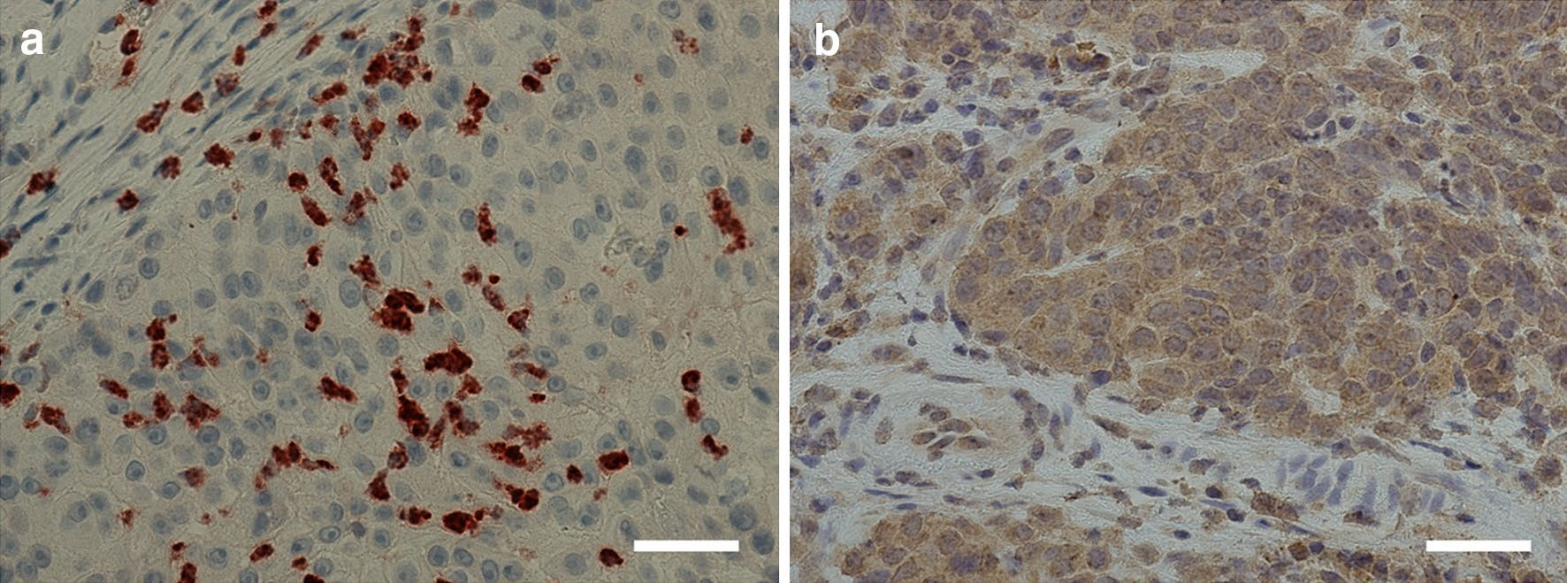
**a** Positive matrix metalloproteinase-8 (MMP-8) immunoexpression in the cells surrounding the tumor tissue. **b** Positive tissue inhibitor of metalloproteinase-1 (TIMP-1) immunoexpression in tumor tissue. *Scale bar* length 50 μm. Magnification × 400

Of the 90 tumors, 84 (93.3%) were available for TIMP-1 IHC. TIMP-1 immunoexpression was detected as cytoplasmic positivity in majority of the tumor cells (*n* = 61, 84.5%, Fig. 1). However, significant differences in the expression between HPV-positive and HPV-negative tumors were not found. Only very few lymphocytes showed TIMP-1 immunopositivity, and thus their immunoscoring was not considered appropriate.

Eighty-three (92.2%) tumors were available for MMP-8 IHC. MMP-8 immunoexpression was absent in tumor cells. However, MMP-8 expression positivity was observed in the inflammatory polymorphonuclear leukocytes adjacent to the tumor in the majority (*n* = 76, 91.6%, Fig. 1) of samples. While a trend towards higher MMP-8 immunoexpression in the inflammatory cells among HPV-negative tumors compared with HPV-positive tumors was observed, the difference was statistically insignificant (*p* = 0.052). The levels of TIMP-1 and MMP-8 immunoexpression and differences between HPV-positive and HPV-negative patients are presented in Table 1.

### TIMP-1 serum levels are several fold higher compared with MMP-8 serum levels

The mean and the median MMP-8 serum levels for HPV-positive patients were 761 pM (Standard deviation [SD] 743) and 762 pM (range, 123–3391 pM), respectively. The mean and the median MMP-8 serum levels for HPV-negative patients were 844 pM (SD 699) and 658 pM (range, 178–3477 pM), respectively.

TIMP-1 serum levels were approximately 10 times higher than MMP-8 serum levels. The mean and the median TIMP-1 serum levels for HPV-positive patients were 8206 pM (SD 4291) and 7054 pM (range, 3274–33,498 pM), respectively. The mean and the median TIMP-1 level for HPV-negative patients were 7869 pM (SD 3128) and 7271 pM (range, 2712–16,322 pM), respectively. However, significant differences in TIMP-1 serum levels or in MMP-8 serum levels were not observed between HPV-positive and HPV-negative patients (Table 1).

### TIMP-1, MMP-8, and survival

The median follow-up time was 37 months (range, 0–62 months). Univariate analysis was first performed for the entire patient cohort. Univariate analysis revealed that differences in TIMP-1 serum levels, stage, age, HPV status, and smoking status were statistically significantly associated with OS. In contrast, differences in TIMP-1 tissue expression, MMP-8 tissue expression, or MMP-8 serum levels did not reach statistical significance (Table 2). A multivariate Cox regression analysis was performed employing all variables that were statistically significant in univariate analysis. Multivariate analysis revealed that high TIMP-1 serum levels were independently associated with poorer OS (adjusted HR 3.6, 95% CI 1.0–12.00, *p* = 0.039) (Table 2).

**Table 2:** Univariate and multivariate Cox regression analysis for overall survival in the whole patient cohort and separately in HPV-positive and HPV-negative patients

	Univariate analysis			Multivariate analysis			Multivariate analysis			Multivariate analysis		
	All patients			All patients			HPV-positive patients			HPV-negative patients		
	HR	95% CI	*p*-value	HR	95% CI	*p*-value	HR	95% CI	*p*-value	HR	95% CI	*p*-value
Age	1.1	1.0–1.1	0.003*	1.1	1.0–1.1	0.004**	1.1	0.9–1.2	0.119	1.0	0.9–1.1	0.249
*Sex*												
Female versus male	1.4	0.6–3.3	0.499									
*Smoking*			0.003*			0.008**			0.016*			0.399
Ex-smoker versus never	1.3	0.3–5.8	0.734	2.0	0.4–9.7	0.380	3.0	0.3–30.0	0.347	2.7	0.2–35.4	0.451
Current versus never	5.5	1.6–18.9	0.007*	6.7	1.8–24.4	0.004**	14.5	1.7–127.6	0.016*	3.1	0.6–16.6	0.176
*T class*												
T3–T4 versus T1–T2	1.3	0.6–2.9	0.578									
*N class*												
N2–N3 versus N0–N1	1.9	0.8–4.9	0.166									
*Stage*												
III–IV versus I–II	3.4	1.5–7.8	0.004*	2.4	0.9–5.9	0.068	1.7	0.3–10.5	0.562	8.7	1.5–50.6	0.017*
*Treatment*												
Sx +− (C)RT versus (C)RT	1.7	0.7–3.8	0.243									
*HPV*												
HPV − versus HPV +	2.6	1.1–6.1	0.024*	1.1	0.4–3.1	0.810						
TIMP-1 serum level	2.7	1.1–6.9	0.037*	3.6	1.1–12.0	0.039*	1.1	0.1–12.1	0.958	14.7	1.8–117.4	0.011*
MMP-8 serum level	1.1	0.66–1.8	0.739									
*TIMP*-*1 immunoexpression*												
2–3 versus 0–1	1.5	0.6–3.8	0.340									
*MMP*-*8 immunoexpression*												
2–3 versus 0–1	1.5	0.6–3.6	0.360									

(*C*) chemo, *CI* confidence interval, *HR* hazard ratio, *MMP-8* matrix metalloproteinase-8, *RT* radiotherapy, *Sx* surgery, *TIMP-1* tissue inhibitor of metalloproteinase-1

Serum TIMP-1 and MMP-8 concentrations are log-transformed. *p* < 0.05*, *p* < 0.01**

### High TIMP-1 serum levels are associated with poorer OS and DFS among HPV-negative patients

Multivariate analysis was performed separately for HPV-positive and HPV-negative groups to evaluate if TIMP-1 serum levels were associated with differences in HRs between these groups (Table 2). High TIMP-1 serum levels were independently associated with poorer OS (adjusted HR 3.6, 95% CI 1.0–117.4, *p* = 0.011) among HPV-negative patients (Table 2). TIMP-1 serum levels did not have any impact on OS among HPV-positive patients.

Additionally, a similar multivariate analysis was performed to evaluate differences in DFS. High TIMP-1 serum levels were independently associated with poorer DFS (adjusted HR 8.7, 95% CI 1.3–57.1, *p* = 0.024) among HPV-negative patients. TIMP-1 serum levels did not have any impact on DFS among HPV-positive patients.

### TIMP-1 serum level cut-off points and survival

**Fig. 2:**
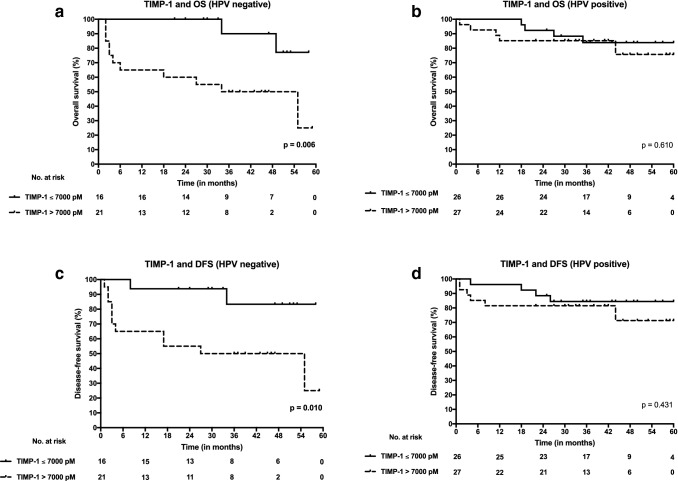
Overall survival (OS) and disease-free survival (DFS) curves according to high (> 7000 pM) and low (≤ 7000 pM) serum levels of tissue inhibitor of metalloproteinase-1 (TIMP-1) both in HPV-positive and HPV-negative OPSCC. **a** TIMP-1 serum level and OS in HPV-negative OPSCC. **b** TIMP-1 serum level and OS in HPV-positive OPSCC. **c** TIMP-1 serum level and DFS in HPV-negative OPSCC. **d** TIMP-1 serum level and DFS in HPV-positive OPSCC

A TIMP-1 serum cut-off value of 7000 pM was found to maximize Youden index. In addition, a TIMP-1 serum level of 7000 pM was close to the median serum level of both HPV-positive and HPV-negative patients. Consequently, it was chosen as an optimal cut-off concentration to discriminate patients into favourable and unfavorable survival groups for further Kaplan–Meier analyses. HPV-negative patients with high TIMP-1 serum levels (> 7000 pM) had significantly poorer OS (*p* = 0.006) and DFS (*p* = 0.010) when compared with patients with lower serum levels (≤ 7000 pM) by Kaplan–Meier method. Similar statistically significant associations were not found in HPV-positive patients. Survival curves drawn by Kaplan–Meier method are presented in Fig. 2.

## Discussion

This prospective study provides new evidence on the potential of TIMP-1 serum levels to serve as an independent prognostic biomarker for OPSCC. TIMP-1 serum levels were found to be a significant independent prognostic marker for OS and DFS in HPV-negative OPSCC patients. Similar results regarding the prognostic value of TIMP-1 serum and plasma levels have also been found in various other cancers [11, 29, 30], including head and neck cancers [27, 28]. However, to the best of our knowledge, this is the first study that is focused on OPSCC only and that compares the prognostic value both in HPV-positive and HPV-negative patients.

The strengths of the present study were the prospective setting with a relatively long follow-up period and availability of both p16 and HPV-DNA status for all tumors. Regarding limitations, MMP-8 and TIMP-1 IHC were not available for all patients and the number of patients was relatively small, which limited more extensive statistical analyses.

TIMP-1 has been reported to have two distinct functions. In addition to directly binding to various MMPs and inhibiting their function, TIMP-1 exerts a specific growth factor function by interacting with the cell surface molecule CD63 and thereby activates intracellular signaling through FAK leading to cell proliferation [24, 25, 31, 32]. It is notable that although TIMP-1 inhibits the proteolytic function of MMP-8, in the present study the serum concentration of MMP-8 was not associated with prognosis. In contrast to other MMPs that in general are associated with promoting cancer invasion and metastasis [9, 17, 18], MMP-8 has been associated with a favorable outcome in various cancers [20, 21].

Although, serum levels of MMP-8 were not associated with survival in the present study, we cannot formally exclude that the association between TIMP-1 and survival is somehow caused by TIMP-1 inhibiting the MMP-8 function. However, our observation that elevated TIMP-1 serum levels, but not MMP-8, are associated with poorer prognosis raises the possibility that the association between TIMP-1 serum concentration and survival is not mediated by the inhibition of MMPs, but instead by interacting with its cell surface receptor CD63 leading to FAK activation. FAK has a key role in immunoevasion and tumor growth and may be a possible target for immunotherapy [14, 47, 48].

In the present study, multivariate analysis revealed that increased TIMP-1 serum levels were independently associated with poorer prognosis in patients with HPV-negative tumors. However, there was also a trend of increased TIMP-1 serum levels and poorer survival in patients with HPV-positive tumors. However, this trend was clearly weaker than in patients with HPV-negative tumors. This weak trend did not reach statistical significance in the present study but might show a stronger effect in a larger patient cohort. Nevertheless, increased TIMP-1 serum levels more strongly associated with poorer prognosis in patients with HPV-negative tumors than in those with HPV-positive tumors. It is possible that the oncogenic changes associated with HPV transformation are sufficiently strong leading to oncogenesis without TIMP-1 up-regulation. The oncogenic changes leading to TIMP-1 up-regulation may be more responsible for cancer progression in HPV-negative tumors. In addition, it is notable that on average HPV-negative tumors have more oncogenic mutations and the mutation profile is different when compared with HPV-positive tumors [49–51]. Additionally, we measured TIMP-1 tumor immunoexpression and this appeared to be positive in the majority of tumor samples. However, in contrast to some previous studies [26, 27, 34] we did not observe an association between different TIMP-1 tumor immunoexpression levels and prognosis despite our observing an association between different TIMP-1 serum levels and survival. There may be several explanations for this. One possibility is that TIMP-1 is rapidly secreted from OPSCC tumor cells and that tissue immunoexpression does not truly reflect the production rate of TIMP-1 in OPSCC tumor cells.

The level of MMP-8 immunoexpression in inflammatory cells appeared to be higher in patients with a HPV-negative tumors than those with HPV-positive tumors. However, the difference was not statistically significant. The differences in the biological and pathophysiological backgrounds of HPV-positive and HPV-negative tumors may explain this phenomenon but a larger patient cohort may be necessary to reach statistical significance.

p16 overexpression, which is characteristic for HPV-positive tumors, is an established independent prognostic factor for OPSCC [6, 8]. Besides p16 and HPV, other comprehensively validated prognostic molecular markers for OPSCC are currently unknown. The survival rates of HPV-negative OPSCC patients have remained generally poor despite of developments in treatment modalities [4, 7, 8]. Thus, there is a clear demand for new prognostic markers, particularly for HPV-negative patients.

## Conclusions

This study provides new evidence for the potential of TIMP-1 serum levels as an independent prognostic biomarker for HPV-negative OPSCC patients. This should be studied in a larger cohort in a multi-center setting to confirm and validate these findings.

## Abbreviations

CD: Cluster of differentiation
CI: Confidence interval
DFS: Disease-free survival
FAK: Focal adhesion kinase
HR: Hazard ratio
IHC: Immunohistochemistry
MMP-8: Matrix metalloproteinase-8
MMPs: Matrix metalloproteinases
OPSCC: Oropharyngeal squamous cell carcinoma
OS: Overall survival
SD: Standard deviation
TIMP-1: Tissue inhibitor of metalloproteinase-1
TIMPs: Tissue inhibitors of metalloproteinases
TMA: Tissue microarray
